# Supplementary material for: Causal Relationship between Aging and Anorexia Nervosa: A White-Matter-Microstructure-Mediated Mendelian Randomization Analysis
Source: Biomedicines. 2024 Aug 16;12(8):1874. doi: 10.3390/biomedicines12081874 (PMC11351342; doi:10.3390/biomedicines12081874)
Supplement: Supplementary file 1 [file biomedicines-12-01874-s001.zip › Supplementary Figure/Figure S4/Singe SNP plot of MR analysis for TL to ubm-b-1934.pdf]

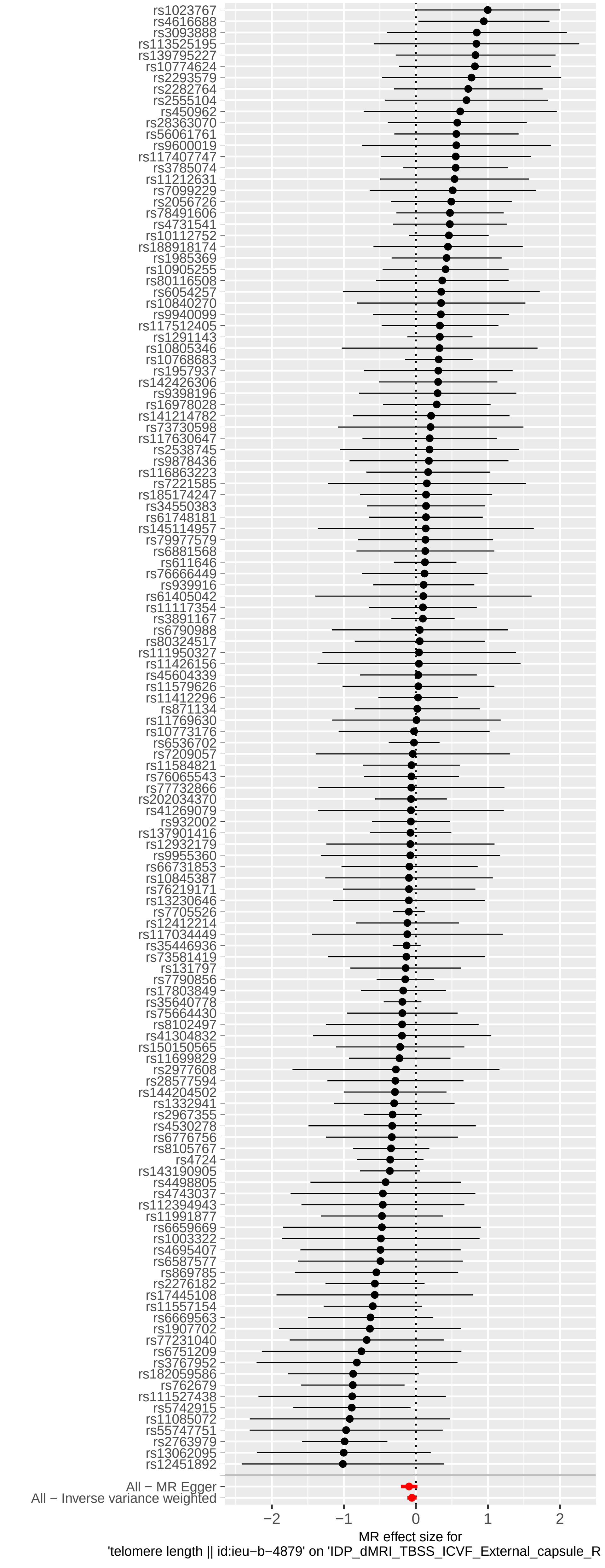
rs1023767
rs4616688
rs3093888
rs113525195
rs139795227
rs10774624
rs2293579
rs2282764
rs2555104
rs450962
rs28363070
rs56061761
rs9600019
rs117407747
rs3785074
rs11212631
rs7099229
rs2056726
rs78491606
rs4731541
rs10112752
rs188918174
rs1985369
rs10905255
rs80116508
rs6054257
rs10840270
rs9940099
rs117512405
rs1291143
rs10805346
rs10768683
rs1957937
rs142426306
rs9398196
rs16978028
rs141214782
rs73730598
rs117630647
rs2538745
rs9878436
rs116863223
rs7221585
rs185174247
rs34550383
rs61748181
rs145114957
rs79977579
rs6881568
rs611646
rs76666449
rs939916
rs61405042
rs11117354
rs3891167
rs6790988
rs80324517
rs111950327
rs11426156
rs45604339
rs11579626
rs11412296
rs871134
rs11769630
rs10773176
rs6536702
rs7209057
rs11584821
rs76065543
rs77732866
rs202034370
rs41269079
rs932002
rs137901416
rs12932179
rs9955360
rs66731853
rs10845387
rs76219171
rs13230646
rs7705526
rs12412214
rs117034449
rs35446936
rs73581419
rs131797
rs7790856
rs17803849
rs35640778
rs75664430
rs8102497
rs41304832
rs150150565
rs11699829
rs2977608
rs28577594
rs144204502
rs1332941
rs2967355
rs4530278
rs6776756
rs8105767
rs4724
rs143190905
rs4498805
rs4743037
rs112394943
rs11991877
rs6659669
rs1003322
rs4695407
rs6587577
rs869785
rs2276182
rs17445108
rs11557154
rs6669563
rs1907702
rs77231040
rs6751209
rs3767952
rs182059586
rs762679
rs111527438
rs5742915
rs11085072
rs55747751
rs2763979
rs13062095
rs12451892
All − MR Egger
All − Inverse variance weighted
−2
−1
0
1
2
MR effect size for
'telomere length || id:ieu−b−4879' on 'IDP_dMRI_TBSS_ICVF_External_capsule_R
